# Intrauterine Growth Restriction: Effects of Physiological Fetal Growth Determinants on Diagnosis

**DOI:** 10.1155/2013/708126

**Published:** 2013-06-23

**Authors:** Kjell Haram, Eirik Søfteland, Radek Bukowski

**Affiliations:** ^1^Department of Obstetrics and Gynecology, Haukeland University Hospital, 5021 Bergen, Norway; ^2^Department of Anesthesia and Intensive Care, Haukeland University Hospital, 5021 Bergen, Norway; ^3^Department of Obstetrics and Gynecology, University of Texas Medical Branch, 301 University Boulevard, Galveston, TX 77555, USA

## Abstract

The growth of the fetus, which is strongly associated with the outcome of pregnancy, reflects interplay of several physiological and pathological factors. The assessment of fetal growth is based on comparison of birthweight (BW) or estimated fetal weight (EFW) to standards which define reference ranges at a spectrum of gestational ages. Most birthweight standards do not take into account effects of physiological determinants of fetal growth. Additionally, gestational age in many standards is based on the menstrual history and is often inaccurate. Fetal growth norms should be based on an early ultrasound estimate of gestational age. Customized standards, which have included only ultrasound-dated pregnancies, seem to be superior to population-based birthweight norms in predicting perinatal mortality and morbidity. Adjustment for individual variation in customized growth curves reduces false-positive diagnosis of IUGR and may lead to a very significant reduction in intervention for suspected IUGR. Customized growth potential identifies better the risk for adverse outcome than the currently used national standards, but customized charts may fail in detecting growth-restricted stillbirth. An individual's birthweight is the sum of physiological and pathological influences operating during pregnancy. Growth potential norms are a better discriminator of aberrations of fetal growth than population, ultrasound, and customized norms.

## 1. Introduction

The assessment of fetal growth is based on comparison of birthweight (BW) or estimated fetal weight (EFW) to standards which define reference ranges at a spectrum of gestational ages [1–3]. EFW and BW are indirect measures of the outcome of primary interest, the intrauterine fetal growth. The growth of the fetus, which is strongly associated with the outcome of pregnancy, reflects interplay of several physiological and pathological factors [4–6]. 

The aim of this study was to review different birthweight standards and evaluate effects of physiological determinants of fetal growth on the ability of the different standards for BW, EFW, and customized growth charts to identify adverse outcomes of pregnancy. 

## 2. Study Design

A non-systematic search in the PubMed was undertaken using terms “birthweight” combined with “gestational age,” “menstrual dating,” “ultrasound dating,” “estimated fetal weight,” “customized growth charts,” “malformation,” “stillbirth,” “family influence,” and “individualized BW ratio,” respectively. We have focused on studies that proposed fetal growth standards which took into account effects of physiological determinants of fetal growth. 

## 3. Determinants of Fetal Size and Growth

### 3.1. Gestational Age Determination: Menstrual versus Ultrasound Dating

Traditional BW norms were usually derived from large databases where gestational age estimates are based on the last menstrual period. The expected size of the fetus will be systematically overestimated when the cycle length is longer than 28 days and systematically underestimated when the cycle length is shorter. Twelve published formula for BPD and femur length (FL) from different centers were reviewed by Mul et al. and systematic and random errors were calculated when they were applied to second-trimester scan measurements in precisely dated pregnancies. Overall, published dating formula performed well in predicting gestational age. The 95% CI interval for BPD was 8.3 days, and for FL 10.2 days. The 95% CI for a second-trimester dating scan performed in the different centers is 8.3 days, with a normal distribution. In the contrast, even by “certain” menstrual history the 95% CI is much wider and skewed towards overestimation of the true gestational age: −9 to +27 days [7]. 

Estimation of gestational age by early ultrasound reduces estimated gestation age in comparison to menstrual dating. Clinically, use of early ultrasound dating instead of menstrual dating is recommended, unless ultrasound is not available [8].

### 3.2. Stillbirths and Congenital Malformations

Most BW standards are based on live births as stillbirths are associated with increased rate of growth impairment [9, 10]. Malformed fetuses as a group also have an increased rate of impaired growth and should be excluded [11]. 

### 3.3. Fetal Sex

Thomson et al. derived BW norms specific for gender and parity from over 50,000 racially homogenous white pregnant women in Aberdeen between 1948 and 1964. They found no significant gender differences in BWs until 34-35 weeks' gestation. After 38 weeks' gestation, males were about 150 g heavier than females [3]. However, a study of pregnancies conceived using assisted reproductive technology, in which gestational age is precisely known, has shown that sex size differences were already established in the first trimester of pregnancy [12].

### 3.4. Parity

BW is also affected by maternal parity. Some population norms are stratified by parity to adjust for a 100–200 g increase in BW between the first and the second birth [13, 14]. 

### 3.5. Race and Ethnicity

Race, ethnicity, fetal sex, parity, maternal weights, and living at high altitude all affect the BW [15]. Norms based on specific racial, ethnic, or regional groups are frequently derived from a relatively small number of individuals and may not well represent larger populations. Their values are therefore limited [15]. The effect of race and ethnicity is also confounded by factors such as poverty and stress. It is therefore uncertain if these differences are due to physiologic, such as genetic factors, or differences in rates of complications of pregnancy affecting fetal growth [16].

### 3.6. Familial Influences

Thomson et al. detected that the maternal size had considerable effect on BW. A baby of a mother which is 170 cm tall and weighing 75 kg was on average almost 750 g heavier than that of a mother being 150 cm tall and weighing 40 kg [3]. 

 A Norwegian study showed that mothers who had experienced two small-for-gestational age (SGA) births (BW < 10th percentile) had lower BW themselves (mean BW 3127 g) compared to mothers who had no previous SGA babies (mean BW 3424 g). The corresponding mean paternal BWs were 3497 g and 3665 g, respectively. Maternal BW seemed to have a stronger influence on offspring BW than that of the father [17]. The infant's BW increased with the height of the mother but was only slightly affected by the father's height [18]. 

A new BW standard incorporating family history has been proposed by Skjaerven et al. in 2000 based on a population of 1.7 million births in Norway between 1967 and 1998 [19]. These population data were arranged into sibships and mother-offspring units through unique personal numbers. The first births were categorized by sex and maternal birthweight and the second births by sex and birthweight of the older sibling. Standards for birthweight per gestational age percentiles differed by >1100 g when the birthweight of an older sibling was considered and by almost 700 g when maternal birthweight was considered [19]. The value of these new standards for birthweight according to gestational age was demonstrated through variation in perinatal mortality. Maternal birthweight and birthweights of previous siblings allow improved predictions of birthweight according to gestational age and should be used for classification of SGA births [19].

**Figure 1:**
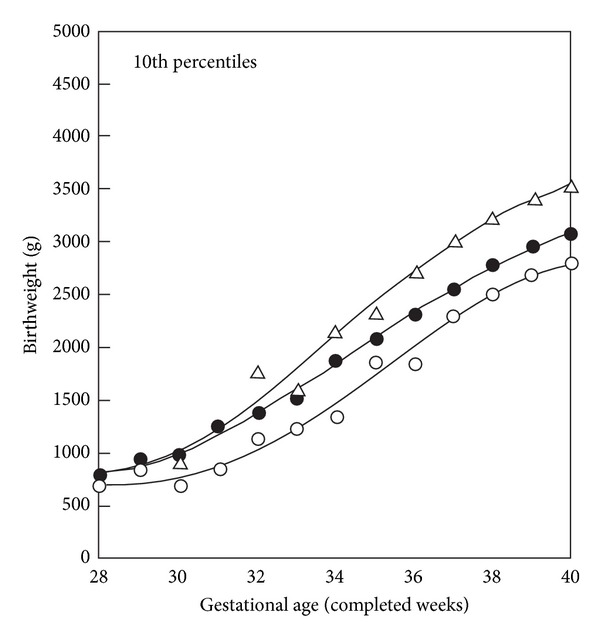
BW percentiles (10th) by gestational age for second singleton births, total, and conditional on weight-by-gestation groups (SGA and LGA) of first births. Smoothed curve values, Norway (1967–1984). ∆: first birth LGA; ●: all second births; ○: first births SGA. The difference between the 10th percentile curves for these subgroups was 720 g at 40 weeks. The figure is modified from Figure  3 in a publication by Skjaerven and Bakketeig [20].

In a publication by Skjaerven and Bakketeig the correlation among offspring's BWs was used in BW norms derived from the Medical Birth Registry of Norway. Gestational age was determined by the last menstrual period. The BW percentiles for second singleton births, conditional upon BW of the first birth, are shown in Figure 1. The difference between the 10th percentile curves for these subgroups was 720 g at 40 weeks' gestation [20]. 

### 3.7. Geographic Effects

In the past 40 years numerous neonatal BW growth curves have been developed following studies in different geographic locations. Some of these studies were hospital based giving rise to potential selection bias particularly due to low [21] or high socioeconomic status [22] of the study population or due to living at high altitude [21]. 

Lubchenco's Colorado study was derived from live-born infants at high altitude [21]. Despite significant limitations, these curves are still widely used in newborn nurseries [23]. Williams et al. established BW norms from over 2 million pregnancies in California. The mortality rates were more strongly associated with the BW than the gestational age at delivery [24].

**Figure 2:**
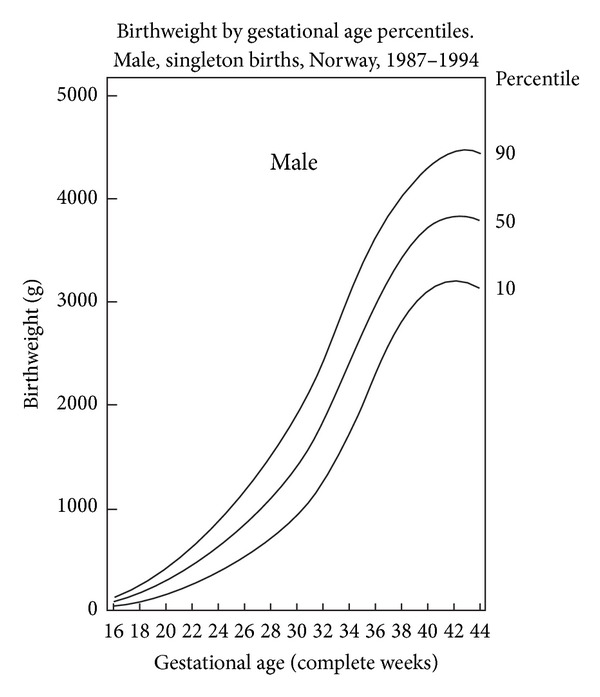
BW by gestational age percentiles, Norway, 1987–1998. Male, singleton births. The figure is modified from Figure  4 in a publication by Skjaerven et al. [10].

**Figure 3:**
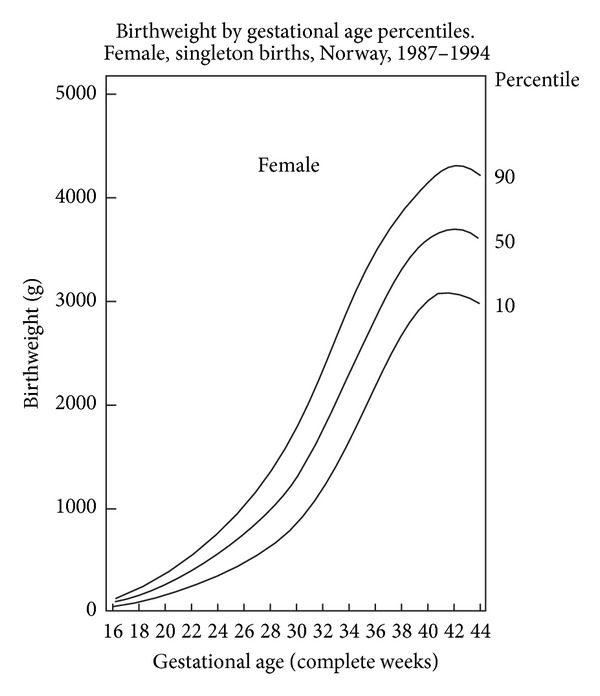
Birthweight by gestational age percentiles, Norway, 1987–1998. Female, singleton births. The figure is modified from Figure  5 in a publication by Skjaerven et al. [10].

 The Birth Registry of Norway records all live births and fetal deaths at ≥16 weeks' gestation. Sex specific BW charts for *nulliparous* and *parous* women have been developed from 1.8 million births from the Birth Registry of Norway between 1967 and 1998 with gestational ages between 20 and 44 completed gestational weeks [10]. These charts have traditionally been used in Norway to define neonatal SGA and intrauterine growth restriction (IUGR) (Figures 2 and 3). 

In Canada sex specific BW norms have been developed by Kramer et al. based on 600,000 singleton live-born infants with ultrasound estimated gestational age of 22 to 43 weeks [25]. Large-for-gestational age (LGA) cutoffs (90th percentile) at low gestational ages are considerably lower than those of existing references, whereas small-for-gestational age cutoffs (10th percentile) *postterm* are higher. For example, compared with the current World Health Organization reference from California [24] and a recently proposed US national reference [26], the 90th percentiles for singleton males at 30 gestational weeks are 1837 versus 2159 and 2710 g, respectively. The corresponding 10th percentiles at 42 weeks are 3233 versus 3086 and 2998 g [25]. Large-for-gestational age cutoffs (90th percentile) at low gestational ages were considerably lower than those of existing references, whereas SGA cutoffs (10th percentile) were higher in *postterm* pregnancies [25]. 

 Graafmans et al. compared BW in seven Western European countries and found substantial differences between countries. Substantial international differences were found in the mode of the BW distribution, which ranged between 3384 g in Flanders and 3628 g in Finland [27]. 

### 3.8. Time Trends in BW

Average BW changes over time, most likely due to improved living conditions. Mean BW has increased substantially over the last quarter of a century in many countries, including USA, Canada, UK, Finland, and India [28, 29]. In Canada an increase of about 200 g of the mean BW at the 40th week of gestation occurred between periods 1970–1972 and 1986–1988 [28]. In Norway there has been a linear increase in BW with a 3.68 g increase in BW per year, that is, around 100 g over a 30-year period until 2000 [10]. In the period between 1989 and 2000 the proportion of newborn >4500 g increased from 3.1 to 4.7% [30]. Therefore, old BW standards require periodic revision. In Norway, the increment in birthweight has now stopped.

### 3.9. Limitations of BW Norms

BW norms usually suffer from three common major limitations. They (1) frequently apply inaccurate dating criteria, (2) establish preterm part of the norms is based on preterm deliveries which frequently are growth restricted [31], and (3) do not account for physiological or pathological determinants of fetal growth [32]. Thus, neonatal BW charts, which derive normal values for BW from preterm fetuses, have lower normal values for preterm gestational ages.

## 4. Examples of Population-Based EFW Norms

**Figure 4:**
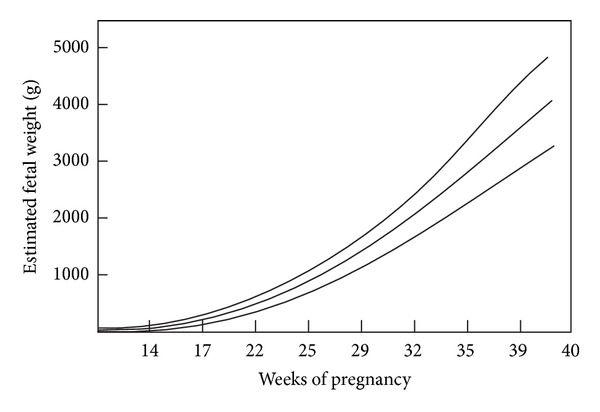
Ultrasonically estimated fetal weights (*n* = 759) based on 86 uncomplicated pregnancies from four Scandinavian centres. The figure is modified from Figure  1 in a publication by Marsal et al. [2].

In Sweden Marsal and coworkers in a cross-sectional study established fetal growth curves based on 759 ultrasonically estimated fetal weights (EFWs) in 86 normal pregnancies from four Scandinavian centres using a formula developed by Persson and Weldener (Figure 4) [2]. 

In longitudinal data each fetus contributes with multiple EFW measurements that together reflect change in size over time [33]. Such longitudinal references for EFW between 20 and 42 weeks have been proposed by Johnsen et al. in a prospective study comprising 634 low-risk pregnancies and a total of 1799 measurement using the formula of Coombs et al. to determine EFW [30]. Maternal body weight, body mass index, and parity did not influence the EFW. Continuous fetal growth was observed until 42 weeks [30]. On the contrary, the study by Skjaerven et al. demonstrated a flattening curve after about 38 weeks' gestation (see Figures 2 and 3) [10]. In a study in a multicultural British population pregnancies dated by ultrasound had mean BW at term higher and showed less flattening growth curve after about 38 weeks than those dated by menstrual history [34].

In the United States new BW curves have recently been proposed in a population of 391,681 infants at 22 to 42 weeks at birth from 248 hospitals within 33 US states between 1998 and 2006. Gender-specific weight, length, and head circumference for gestational age curves were created, and their distributions were validated in a separate sample [35]. The SGA and large-for-gestational age classifications, using the Lubchenco curves, differed significantly from those new US curves at each gestational age [35]. The BWs curves publised by Skjaerven et al. were largely similar to those new US norms; however, the 10th, 50th, and 90th percentiles were slightly higher [10].

## 5. Individualized Norms Accounting for Physiological Variables

### 5.1. Customized Fetal Growth Charts

Gestational age, fetal sex, maternal weight and height, race and ethnicity, parity, and prepregnancy body mass index were all found to be determinants of fetal growth. Moreover, several pathological factors are associated with intrauterine growth restriction (IUGR) due to conditions like hypertension, smoking, preterm delivery, *diabetes mellitus*, and preterm rupture of membranes [4, 36].

**Figure 5:**
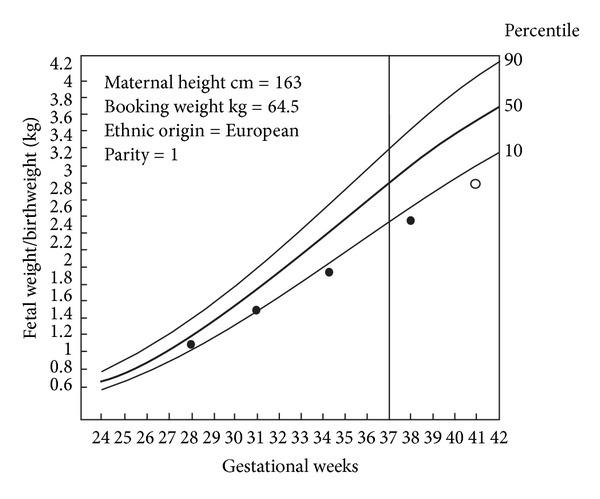
Example of customized growth charts. Chart representing expected population average growth in pregnancy, mother with of height 163 cm and booking weight 64.5 kg, European ethnic group, parity 1. The line through 37 weeks denotes onset of “term.” On the left *y*-axis fetal weight/birthweight. The curves represent the 50th, 90th, and 10th percentiles for the individually adjusted limits of fetal weight/birthweight. Serial weight measurements (●) leading to a birthweight of 3 kg (○). The figure is modified from Figure  1, in a publication by Mongelli and Gardosi [57, page 846], with permission.

**Figure 6:**
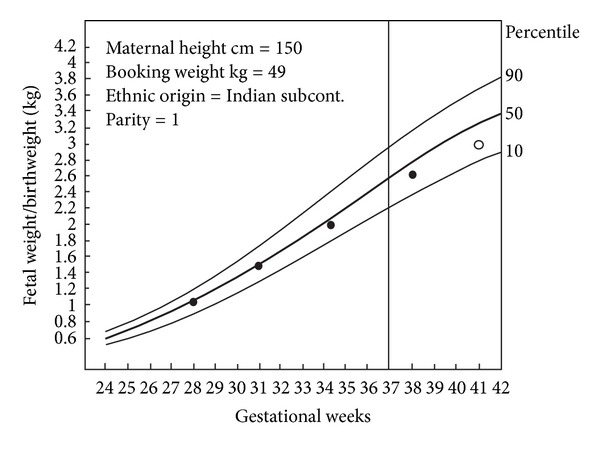
Examples of customized growth carts. Chart representing expected population average growth in pregnancy, with mother of height 150 cm, booking weight 49 kg, Indian subcontinent ethnic group, parity 1. The line through 37 weeks denotes onset of “term.” On the left *y*-axis fetal weight/birthweight. The curves represent the 50th, the 90th, and the 10 percentiles for the individually adjusted limits of fetal weight/birthweight. Serial weight measurements (●) showing normal fetal growth leading to a birthweight of 3 kg (○). This figure is modified from Figure  2, in a publication by Mongelli and Gardosi [57, page 847], with permission.

Gardosi et al. have proposed an antenatal growth norm, individually adjusted or “customized” for maternal height, weight in early pregnancy, parity, ethnic group, and fetal sex (Figures 5 and 6). In short, the optimal birthweight at term is modulated by linear regression, which takes into account the biologic characteristics [4, 37]. 

Customized centiles based on individual fetal growth potential enhance the ability to differentiate between physiological and pathological smallness. The standard has to exclude pathology, such as hypertensive disease in pregnancy or *diabetes mellitus*. The most common example is smoking, which is known to affect birthweight in a dose-related relationship up to a deficit of 250 g at term [38]. The use of the growth potential refines the distinction between physiology and pathology in two ways. Firstly, it identifies an additional group, up to one third of more cases than the original “SGA” population [39], which are not recognized as SGA by conventional, population-based birthweight centiles. This “unrecognized SGA” group could, for example, include SGA babies which were born preterm and were not recognized as SGA by the population-based neonatal weight standard [40], or it could include babies of large mothers with birthweights which are within the population average, but small compared to the customized growth potential [38].

Most published customized standards have included only ultrasound-dated pregnancies and have been shown to be superior to population-based BW norms in predicting perinatal mortality and morbidity. Clausson et al. validated customized norms using the Swedish Birth Registry. In a cohort of 326,377 births they calculated percentiles of BW based on population norms adjusted for sex and gestational age and also a customized BW standard. In comparison to a population based BW standard, customized norms increased identification of stillbirth, neonatal death, and five-minute Apgar score under four [39]. 

Adjusting BW standards for maternal and infant characteristics improved prediction of adverse pregnancy outcomes also in a US population [41] and in a Dutch study of 3,217 high risk pregnancies [42]. In a case-control study from Texas comprising 354,205 spontaneous uncomplicated preterm deliveries the use of customized percentiles improved the identification of pregnancies with increased risk of perinatal death [31]. 

It has been shown that when adjusted or customized percentiles were applied in antenatal care, 28% of babies conventionally designated as SGA (<10th percentile) and 22% of those designated as large-for-gestational age (>90th percentile) were reclassified to be within normal limits [43].

In a study published in 2006 using customized charts for assessment of fetal growth in Blackburn, where 35% were non-Caucasian (mainly Indo-Pakistani), it was shown that introduction of such standard could lead to a very significant reduction in intervention for suspected IUGR [44]. 

### 5.2. Customization and Stillbirth

In an analysis of stillbirths in Oslo, 52% of “unexplained” *antepartum* fetal deaths (without any postmortem findings) IUGR were defined by customized curves (<10th percentile) [45]. Stillbirth and perinatal mortality studies lack precise estimate of time of death, and therefore the duration of pregnancy is a limiting factor. The usefulness of customized charts was questioned as they failed to perform better than unadjusted charts in detecting growth-restricted stillbirth [46]. This is likely due to uncertainty of gestational age at time of death in stillbirths which causes misclassification of pregnancies and masks the effects of physiologic factors. Clearly, better designed studies are needed especially ones evaluating primary outcomes of stillbirth and perinatal mortality. Those studies should properly account for time of death in calculation of gestational age so as not to inflate the rate of growth restriction. Such high quality evidence is required to demonstrate that customized fetal norms perform better than unadjusted standards before they are implemented in practice [46].

In a study comparing customized and population-based BW standard conducted in a maternity hospital in France from 1997 to 2002, comprising 56,606 births, it was found that, by using customized standards, 2.7% of births were reclassified as SGA. Compared with non-SGA births, these newly detected SGA neonates showed an increased risk of stillbirth (odds ratio (OR) 4.52, 95% CI 2.47–8.14) and perinatal death (OR 2.60, 95% CI 1.62–4.15) [47]. 

In a study by Odibo et al. SGA defined by the customized growth chart was compared with a population-based growth chart for USA [48]. The cases additionally identified as SGA by the customized method had a significantly increased risk of adverse outcome. The sensitivity and specificity of those identified as SGA by customized method only for detecting pregnancies at risk for stillbirth was 32.7% (95% CI 27.0–38.8%) and 95.1% (95% CI 94.7–95.0%) versus 0.8% (95% CI 0.1–2.7%) and 98.0% (95% CI 97.8–98.2%) for those identified by only the population-based method, respectively [48].

A retrospective, cohort study was undertaken to estimate the impact of adding ultrasound biometric parameters to customized chart for the prediction of intrauterine death [48]. After exclusion of pathology, 59,016 births were identified. Coefficients for significant physiological and pathological variables affecting fetal growth were derived using backward stepwise multiple regression (Cust-chart). The same process was repeated including second-trimester biometric parameters BPD, head circumference, femur length, and abdominal circumference in the regression models (Cust-plus-USS-chart). The association between SGA <10th centiles pregnancies, defined using the two customized charts or our population-based growth chart (Pop-chart) and intrauterine fetal death (IUFD), was compared. The OR for the association between SGA defined by the three charts and IUFD was 7.0 (4.5–11), 6.5 (4.2–10.2), and 2.4 (1.6–3.6) according to the Cust-chart, Cust-plus-USS-chart, and Pop-chart, respectively. It was concluded that customized charts are more efficient in identifying pregnancies at risk for intrauterine fetal death than population-based charts. However, adding second-trimester ultrasound biometric parameters to the customized model did not improve the prediction of intrauterine fetal death compared with using maternal characteristics only [48].

### 5.3. The Value of Customized BW Standard Is Controversial

When testing the hypothesis that customized growth curves are superior to population-based curves, several reports have suggested that this methodology more accurately predicts morbidity and mortality at the lower end of BW [49] and that the apparent benefits are more likely to have been derived from incorporation of EFW-based reference values at preterm ages than adjustment for maternal characteristics [50]. In a population-based study comprising 782,303 singletons ≥28 weeks of gestation born in 1992–2001 to Nordic mothers the customization led to a large artificial increase in the proportion of SGA infants born preterm. It was claimed that the large increase in perinatal mortality risk among infants classified as SGA on customized standards was largely an artefact due to inclusion of more preterm births and that the customization technique by Zhang et al. overestimates the risk of fetal and neonatal mortality [51]. Customized percentiles were unable to distinguish between pathological and physiological influences of maternal characteristics on BW [50] and should therefore not be accepted as a “package deal” [52]. However, Figueras and Gardosi in a recent publication concluded that there is good quality evidence to recommend adjusting BW for maternal and pregnancy variables to improve the distinction between constitutional and pathological smallness [4]. 

### 5.4. Calculation of Customized Fetal Weight Percentiles with Anthropometric Features of IUGR

Owen et al. have calculated customized centiles adjusted for maternal height, booking weight, ethnic origin, together with fetal sex, gestational age, and birth order in a population of 313 pregnant women in Dundee (UK). Three separate neonatal anthropometric measures were used to define IUGR: a subscapular or triceps skinfold thickness <10th percentile, a ponderal index <25th percentile, and a mid-arm circumference to occipitofrontal circumference ratio less than one standard deviation. Adjustment for individual variation in customized growth curves reduces the false-positive diagnosis of IUGR which moderately useful in the identification of neonates with low skinfold thickness and low ponderal index [43].

Owen et al. also have calculated third-trimester customized fetal weight percentiles in infants born with anthropometric features of IUGR and compared these to ultrasound estimates of the growth velocity in 258 normal fetuses [53]. They concluded that customized percentiles in the late third trimester were less accurate than calculated growth velocity in predicting such defined IUGR [53].

### 5.5. Individualized BW Ratio

The individualized BW ratio is a measure of the difference between the actual BW of an infant and a predicted BW calculated from the relative contributions of gestational age, maternal weight, infant sex, maternal height, parity and ethnic origin. Using an individualized BW ratio less than the 10th percentile as cut-off results in 25% of those less than the 10th percentile of BW for gestation being reclassified as normally grown. The individualized birthweight ratio combines the simplicity of birthweight measurement with the accuracy of clinical measurements in the identification of IUGR [54]. 

### 5.6. Individualized Fetal Growth Trajectory

An alternative approach to individualization of fetal growth is to calculate an individual fetal growth curve for each fetus based on fetal growth trajectory in the second trimester of pregnancy. Such approach has been proposed by the Rossavik growth model based on growth patterns before 26 weeks' gestation of head and abdominal cubes [55]. This procedure provides an individualized assessment of growth. BW as well as weight estimates can be predicted with a high degree of accuracy (±12%) in normal growing fetuses [55]. However, this method requires at least two ultrasound scans during the second trimester in order to calculate growth velocity and assumes that restriction of fetal growth does not occur before then [5]. 

### 5.7. Growth Potential Norms

An individual's BW is the sum of physiological and pathological influences operating during pregnancy [56]. Recently, fetal growth potential norms have been designed in a prospective cohort of 38,033 singleton pregnancies dated by first-trimester ultrasound [56]. Nineteen physiologic factors, associated with maternal characteristics and early placental function, were associated with BW among normal pregnancies. Growth potential norms correctly classified significantly more pregnancies than population, ultrasound, or customized norms among all complicated pregnancies: 26% (growth potential norms), 18% (population norm), 19% (ultrasound norm), and 23% (customized norm), respectively. Growth potential norms also classified more correctly pregnancies with diabetes or hypertensive disorders and neonatal complications [56]. Thus, growth potential norms are a better discriminator of aberrations of fetal growth than population, ultrasound, and customized norms. They were designed using precise first-trimester estimation of gestational age, the most powerful determinant of BW, and account for first- and second-trimester variations in fetal growth. The physiological ranges of continuous determinants of BW used in those norms, such as weight, height, body mass index, and concentrations of placental hormones, are based on their ranges observed in normal pregnancies rather than arbitrary chosen [56]. 

## 6. Conclusions

Race, ethnicity, fetal sex, parity, maternal weights, and living at high altitude all affect BW. Standards of BW should be derived from large, unselected, uncomplicated, and ultrasound-dated populations representative of the targeted populations. FFW standards should be derived from longitudinal using a formula to calculate EFW. Bukowski et al. have proposed an antenatal growth norm, individually adjusted or “customized” for maternal height, weight in early pregnancy, parity, ethnic group, and fetal sex. Customized centiles based on individual fetal growth potential enhance the ability to differentiate between physiological and pathological smallness. The standard has to exclude pathology, such as hypertensive disease in pregnancy or *diabetes mellitus*. Effects of the physiologic determinants of BW should be considered, keeping in mind that some of these factors may not have independent effects on fetal growth. In a study severe impairment of an individually calculated growth potential was found to be strongly associated with neonatal encephalopathy. Growth potential norms appear to better discriminate aberrations of fetal growth than population, ultrasound, and customized norms. To detect such cases norms have to be based on first-trimester ultrasound dating [55]. The accuracy of individualized growth potential norms may also be improved by better identification of a *cut-off* percentile to define growth impairment based on pregnancy outcome rather than arbitrary. Further studies are needed to further improve existing norms. Interventional trials should also be performed to validate the clinical value of the knowledge of the fetal growth.

## Abbreviations

BPD: Biparietal diameter
BW: Birthweight
95% CI: 95% confidence interval
EVF: Estimated fetal weight
FL: Femur length
LGA: Large for gestational age
IUFD: Intrauterine fetal death
IUGR: Intrauterine growth restriction
SGA: Small-for-gestational-age
OR: Odds ratio.
